# Influence of Organic Matter on the Sorption of Cefdinir, Memantine and Praziquantel on Different Soil and Sediment Samples

**DOI:** 10.3390/molecules27228008

**Published:** 2022-11-18

**Authors:** Dragana Mutavdžić Pavlović, Kristina Tolić Čop, Helena Prskalo, Mislav Runje

**Affiliations:** 1 Department of Analytical Chemistry, Faculty of Chemical Engineering and Technology, University of Zagreb, 10000 Zagreb, Croatia; 2 Pliva Croatia TAPI R&D, 10000 Zagreb, Croatia

**Keywords:** sorption, memantine, cefdinir, praziquantel, organic matter, distribution coefficient

## Abstract

Pharmaceuticals are known for their great effects and applications in the treatment and suppression of various diseases in human and veterinary medicine. The development and modernization of science and technologies have led to a constant increase in the production and consumption of various classes of pharmaceuticals, so they pose a threat to the environment, which can be subjected to the sorption process on the solid phase. The efficiency of sorption is determined by various parameters, of which the physicochemical properties of the compound and the sorbent are very important. One of these parameters that determine pharmaceutical mobility in soil or sediment is the soil–water partition coefficient normalized to organic carbon (*K*_oc_), whose determination was the purpose of this study. The influence of organic matter, suspended in an aqueous solution of pharmaceutical (more precisely: cefdinir, memantine, and praziquantel), was studied for five different types of soil and sediment samples from Croatia. The linear, Freundlich, and Dubinin–Raduskevich sorption isotherms were used to determine specific constants such as the partition coefficient *K*_d_, which directly describes the strength of sorbate and sorbent binding. The linear model proved to be the best with the highest correlation coefficients, *R*^2^ > 0.99. For all three pharmaceuticals, a positive correlation between sorption affinity described by *K*_d_ and *K*_oc_ and the amount of organic matter was demonstrated.

## 1. Introduction

In the last 30 years, pharmaceuticals have become of great interest to many scientists. In fact, they are suspected of adversely affecting human and animal health because, due to a significant increase in production and consumption, they now ultimately enter the environment in large quantities, directly or indirectly, through various pathways. The physico-chemical properties (good water solubility and low degradability) of some of these substances allow them to pass through natural filters and water treatment plants. They are present in the environment either in the unchanged form of the parent compound or in the form of the corresponding active metabolites. In this way, they affect drinking water supplies, groundwater quality and the life of terrestrial and aquatic organisms. Since analytical techniques have not been able to achieve lower sensitivity (at the nano/micro scale) for many years, there is still a lack of information on the fate of many contaminants such as pharmaceuticals in the environment [1,2,3].

Environmental contamination by pharmaceuticals depends on several parameters, including the type and amount of a particular pharmaceutical. The transfer of pharmaceuticals from the treated area to surface water is primarily influenced by their water solubility, degradability, photostability, acid dissociation constant (*pK*_a_), partition coefficient (*K*_d_), partition coefficient of organic carbon in water (*K*_oc_), and partition coefficient of octanol in water (*K*_ow_). Weather conditions and soil/sediment composition have a great influence on environmental behavior [1,4,5].

Sorption is an important process because it can significantly influence and control the fate and effects of organic compounds in the environment [6]. To describe the sorption affinity of pharmaceuticals to a solid, the partition coefficient *K*_d_ (L/kg) is commonly used. It is determined experimentally from the slope of the linear isotherm, representing the ratio of solid (*q*_e_) and liquid (*C*_e_) phases according to the following equation [7]:

(1) $$q_{e}=K_{d}\times C_{e}$$

In addition to the linear isotherm, the amount of pharmaceutical sorbed onto the solid samples can also be determined by Freundlich (Equation (2)) and Dubinin–Raduschkevich (Equation (3)) isotherms according to the following equations [5,8]:

(2) $$log\;q_{e}=logK_{F}\times \frac{1}{n}$$

(3) $$q_{e}=q_{m}exp\left(\beta \varepsilon^{2}\right)$$

where *q*_e_ and *C*_e_ represent the sorbed amount of the pharmaceutical (µg/g) and the final concentration at equilibrium (µg/mL); *K_F_* is the Freundlich sorption coefficient ((μg/g)/(μg/mL)^1/*n*^); and *n* is the dimensionless Freundlich exponent describing the deviation from linearity between the sorbed pharmaceutical and the residual concentration at equilibrium; *q_m_* is the maximum sorption capacity (µg/g); *β* is a constant related to the average sorption energy (mol^2^/kJ^2^), and *ε* is the Polany potential obtained from equation (Equation (4)):

(4) $$\varepsilon =RTln\left(1+\frac{1}{C_{e}}\right)$$

The Freundlich isotherm is used to test whether the sorption process occurs on the heterogeneous surface of the sorbent at active sites with different levels of adsorption energy [9]. Since the parameter *n* indicates the intensity of sorption, its values can be used to infer the sorption. For example, when *n* < 1, the isotherm takes a concave shape because the free energy of binding between the sorbed molecules and the sorbent is lower. In the case of *n* = 1, the isotherm is linear because the free energy is constant at all concentrations of the tested molecules, and in the case of *n* > 1, the isotherm is convex because the free energy is higher as more molecules are sorbed near the adsorbent [10]. By determining the parameters of the Dubinin–Raduschkevich isotherm, information about the reaction mechanism and the nature of sorption can be obtained. If the value of the free energy of sorption E < is 8 kJ/mol, it can be assumed that physisorption predominates, in contrast to chemical sorption where the values of the free energy of sorption are between 8–16 kJ/mol [11,12].

Since the *K*_d_ value depends on the organic carbon (or organic matter) content, it is better to use the partition coefficient *K*_oc_, normalized to organic carbon, to model the distribution in the environment under consideration and obtain information about the potential risk of pharmaceuticals [4,13,14]. The relationship between *K*_d_ and *K*_oc_ coefficients was defined by the following equation [15]:

(5) $$K_{oc}=\frac{K_{d}}{\%OC}\times 100;$$

(6) $$\frac{\%OM}{\%OC}=1.724$$

Organic matter in soils and sediments is a complex, heterogeneous material. In addition to the familiar humic fractions such as humic acid (HA), fulvic acid, and humin, it also consists of a considerable amount of particulate organic matter. This organic matter is usually not bound to other soil/sediment components such as minerals, but the interaction of the particulate organic matter that accumulates on the soil/sediment surface with contaminants is important [16]. The situation is somewhat different for sediments because they are in contact with water most of the time. The water in contact with the sediment washes out many substances, including the organic matter, especially from the part that is constantly in the water. In sediments exposed to climatic influences due to water withdrawal during certain dry periods, a higher content of organic matter is observed. This content is still lower than that in soils from the same geographical area, but its importance is the same. Humic substances in sediments and organic particles in the water phase play an important role in the flocculation and accumulation of hydrophobic compounds, thus affecting the fate and dispersion of contaminants in the environment [17,18]. In addition, suspended organic matter can allow the formation of new active sorption sites, while higher concentrations of dissolved humic acid can cause desorption of pharmaceuticals from soil and facilitate their transfer to water [19].

However, few studies have investigated the interaction between organic matter (OM) and pharmaceutical agents such as diclofenac [20], sulfamethazine [21], and tetracyclines [16,22] and polycyclic aromatic hydrocarbons (PAH)/chlorophenol [17]. Additionally, Guo and coworkers [23] studied the sorption of tylosin (TYL) and sulfamethazine (SMT), two ionizable antibiotics commonly used in agriculture as veterinary therapeutics and growth promoters, to solid matter HA. The high sorption of these compounds to soils and sediments is often associated with a high HA content. OM is usually represented in the literature by humic acids, as these are commercially available. The literature emphasizes that the sorbing efficiency is related to the hydrophobicity of the sorbate, and that the interaction of HA with hydrophobic organic matter is mainly due to π–π interactions, hydrophobic effects and hydrogen bonding [24].

In this paper, three pharmaceutical target compounds from different therapeutic classes were selected. Praziquantel is a low-toxicity anthelmintic pharmaceutical known for the treatment of Schistosoma worms infections and other novel tropical diseases [25,26]. Memantine, an *N*-methyl-D-aspartate (NMDA) receptor antagonist is used to alleviate symptoms associated with Alzheimer’s disease [27]. Cefdinir is a cephalosporin antibiotic used to treat urinary tract infections, osteomyelitis, and meningitis. Its presence in wastewater has been confirmed at high concentrations (125–175 mg/L), making it and other cephalosporins a group of emerging contaminants [28]. Although environmental data were found only for cefdinir to the authors’ knowledge, all three pharmaceuticals tested are widely distributed and are expected to be present in the environment. Ribeiro and coworkers [29] have drawn attention to this problem with respect to cephalosporins, but also to other widely used antibiotics [30], particularly with respect to their behavior in different soil types [31]. The fact that cephalosporins can be sorbed well to soil and sediment particles is shown by the sorption potential of the aqueous cephalexin solution to interact with activated charcoal and grapevine, where recovery rates of more than 80% have been achieved with this method of removal from an aqueous medium.

The aim of this paper was to experimentally determine how the influence of organic matter affects the sorption affinity of all three pharmaceuticals by using natural soil and river sediment samples. These samples possessed different properties depending on the sampling location from which they were taken in different Croatian areas. Linear, Dubinin–Radushkevich and Freundlich isotherms were used to describe the sorption tendency. The mechanism of sorption was defined by kinetic models: Lagergren’s pseudo-first-order pseudo-model, second-order pseudo-model and intraparticle diffusion model. The thermodynamic parameter standard Gibbs energy (ΔG°) was calculated from the estimated *K*_d_ values at 25 °C. The results of the present study may contribute to a better understanding of the mobility and fate of the studied pharmaceuticals in ecosystems.

## 2. Materials and Methods

### 2.1. Materials

The following high-purity (>99%) pharmaceuticals were used in this study: cefdinir (CEF), memantine (MEM), and praziquantel (PRAZ). CEF and MEM were purchased from Sigma-Aldrich (Steinheim, Germany), whereas PRAZ was purchased from Genera d.d. (Kalinovica, Croatia). The formulas of the studied pharmaceuticals and their physico-chemical properties are listed in Supplementary Materials Table S1. Stock solutions of each pharmaceutical were prepared separately by dissolving a certain amount of the pharmaceutical into an appropriate volume of 0.01 M CaCl_2_ to obtain working standard solutions (2.0; 1.0; 0.5; 0.3; 0.2 and 0.1 mg/L) by serial dilution. The working solutions of the studied pharmaceuticals 2.0 mg/L also contain a maximum of 1% methanol for better dilution of the studied pharmaceuticals in aqueous solutions. All solutions were stored in a way that protected them from light at 4 °C. Acetonitrile HPLC grade was supplied by Baker (Deventer, The Netherlands) and formic acid was purchased from Merck (Darmstadt, Germany).

Humic acid, manufactured by Sigma-Aldrich (Steinheim, Germany), was obtained as a dry formulation powder. A total of 1 mg/L humic acid solution was prepared by dissolving 0.5 mg humic acid powder in 500 mL of 0.01 M CaCl_2_. The solution was placed in an ultrasonic bath to ensure complete dissolution of humic acid. The pH of this humic acid solution was checked to establish that is was 7.00, and adjusted accordingly if there were minor deviations.

### 2.2. Sediment and Soil Samples

Experiments were performed with five samples of river sediment and five natural soil samples. The sediment and soil samples were collected in six different Croatian regions: Sisak-Moslavina County (sediment 1—Glina River, sediment 2—Lonja River, sediment 3—Petrinjčica River), Zadar County (soil 1—Bruvno and soil 2—Gračac), Brod-Posavina County (soil 3—Dolina and soil 4—Ljupina), the Požega-Slavonia County (sediment 4—Pakra River), Osijek-Baranja County (soil 5—Josipovac) and the Primorsko-Goranska County (sediment 5—Studena River). All samples were collected in an area far from human activities (no farms or factories), so the samples should not contain pharmaceuticals, especially those tested. Major or minor differences in their physicochemical properties give a clear picture of which of the physicochemical parameters is crucial for the sorption of a particular tested pharmaceutical.

Samples of both sediments were collected during the summer by hand-held device, and with a trowel below the aqueous layer when the water level was lower, which greatly simplified the overall sampling procedure. All collected solid samples were dried, ground, sieved through a 2 mm sieve, and characterized in the previously described manner [13].

Table S2 shows the physicochemical properties of the sorbent samples used.

### 2.3. Batch Sorption Experiments and Data Analysis

All batch sorption experiments of the tested pharmaceuticals on river sediment and natural soils were performed according to the OECD 106 procedure [32]. The procedure is performed in triplicate by shaking with a laboratory shaker (Innova 4080 Incubator Shaker, NewBrunswick Scientific, Edison, NJ, USA), which allows continuous contact between the sediment or soil samples and the solutions of the tested pharmaceuticals. To avoid photolytic degradation, shaking is performed in the dark, and to avoid microbiological activity, all samples are sterilized beforehand.

To determine the contact time required to reach sorption equilibrium, preliminary experiments were performed by shaking soil/sediment samples with solutions of the tested pharmaceuticals at different time intervals (10, 20, 30, 40 and 50 min and 1, 2, 4, 6, 12, 18, and 24 h) at 25 °C. Derived from the preliminary experiments, a time period of 24 h was chosen for all further experiments. These results also form the basis for gaining knowledge about sorption kinetics.

The same sorption procedure is performed for each of the tested pharmaceuticals. The procedure consists of adding 10 mL of the solution of one of the tested pharmaceuticals at a known concentration (0.1–2.0 mg/L) in 50 mL of laboratory glassware to 1 g of air-dried sediment or soil samples. Based on the preliminary tests, the prepared suspension was shaken in a shaker (at 200 rpm) for 24 h at controlled temperature conditions (adjusted at 25 °C), filtered through 0.45 µm syringe filters, and added to HPLC vials. To investigate the effect of organic matter on the sorption of studied pharmaceuticals, experiments were performed with four concentration levels of humic acid (0; 1; 10 and 100 mg/L). Therefore, 9 mL of the prepared humic acid solution were added to all the laboratory glassware prepared for the series of experiments with a humic acid concentration of 1 mg/L of solution, as previously mentioned (the total volume of the solution in the laboratory glassware was 10 mL).

When investigating the effects of higher humic acid concentrations (10 mg/L and 100 mg L) on the sorption of the tested pharmaceuticals, the procedure is somewhat different. Since humic acids are sparingly soluble, it is not possible to prepare solutions containing 10 and 100 mg/L. Therefore, the required amount of humic acids is added to 10 mL of the pharmaceutical solution (which is already in contact with the soil and sediment) to obtain the desired concentration of humic acids. For these experiments, 0.10 mg (for 10 mg/L) and 1.00 mg (for 100 mg/L) of humic acid were dissolved in 10 mL of one of the six concentrations of each pharmaceutical. All experiments were performed in 0.01 M CaCl_2_ solution at the initial pH of pharmaceutical under study (in the case of CEF this is pH 6.0, in the case of PRAZ pH 6.5, and in the case of MEM pH 7.0).

The residual concentration of the pharmaceutical (CEF, MEM and PRAZ) in the remaining liquid phase after sorption was analyzed by the UHPLC-MS (Agilent 6490 coupled with Agilent Infinity UHPLC system Triple Quadrupole Mass Spectrometer, Santa Clara, CA, USA) with electrospray ionization according to the method described previously [33].

## 3. Results and Discussion

### 3.1. Effect of Contact Time and Initial Concentration of Pharmaceuticals

To determine the sorption coefficient, it is first necessary to determine the time within which the maximum sorption of the tested compounds can be achieved for the soil and sediment samples tested. Since the initial concentration of the tested compounds plays an important role, three different concentrations (0.1, 0.5 and 2.0 mg/L) were prepared for each tested pharmaceutical. Each of these solutions were contacted with the tested soil and sediment samples and shaken for different time intervals (10, 20, 30, 40, 50 min and 1, 2, 4, 6, 18 and 24 h). Some of the results can be seen in Supplementary information, Figure S1. Sorption kinetics of CEF, PRAZ, and MEM can be divided into two phases: “fast” and “slow” sorption [34]. During the first hours (at the lowest concentration) or 6 h after shaking (at the higher concentrations), intense sorption of the pharmaceuticals takes place on the tested samples. After the mentioned times, the sorbed amounts do not change in all cases until the final equilibrium is reached due to the gradual saturation of the sorption active sites [34]. Although in all cases the maximum sorption is reached after 18 h of shaking, to simplify the experiments all further experiments were performed with 24 h of shaking. The lowest percentage of sorption for CEF and PRAZ solutions is obtained at the lowest concentration (0.1 mg/L), while in the case of MEM the lowest sorption is obtained at the highest concentration (2.0 mg/L). For CEF sorption, the percentage of sorption in both samples shown changes only slightly with the change in concentration, while for PRAZ sorption the sorption effect is significant or at least more pronounced compared to CEF sorption.

### 3.2. Sorption Isotherms

In the determination of sorption coefficients, concentrations of the studied pharmaceuticals in the range of 0.1 mg/L to 2 mg/L were used, corresponding to a range of 1 to 20 mg/kg soil/sediment. The range of concentrations studied is consistent with the average concentrations found in studies of a similar nature (about 0.2–20 mg/kg) [21,35,36], although the ranges in environmental samples are much larger (for sediment 0.02–285 mg/kg, for soil 0.034–530 mg/kg) [37,38]. CEF, MEM, and PRAZ are relatively unexplored in environmental samples, and it is difficult to say whether the concentration chosen for this study corresponds to their actual concentration in the environment. Nevertheless, the lowest concentrations were chosen to allow for analytical detection [39].

**Figure 1:**
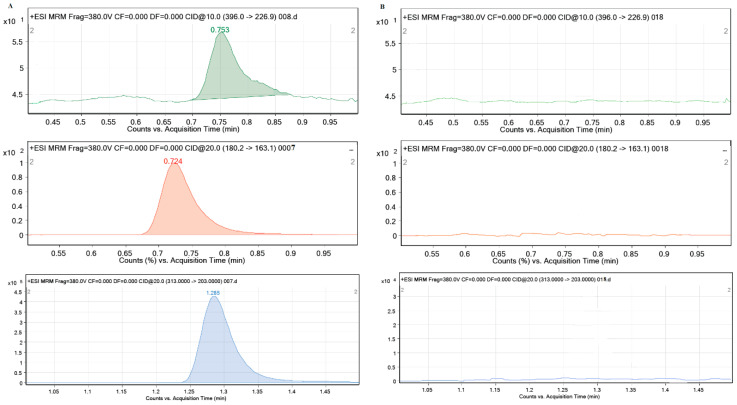
Characteristic MRM mass spectra of (**A**) standard solution of CEF: 396.0→226.9; MEM: 180.2→163.1; PRAZ: 313.0→203.0 and (**B**) investigated sediment matrix (Glina).

During the performance of all experiments, the stability of the tested pharmaceuticals in solution and their possible sorption on the walls of the test vessels were investigated. For this purpose, the control samples CEF, MEM and PRAZ were used with the highest concentrations (2 mg/L) in 0.01 M CaCl_2_ and analyzed for LC-MS/MS. The obtained results showed that all pharmaceutical products are stable in aqueous solutions for the required time and no decrease in their concentration was observed during the performance of all experiments, which means that they were not sorbed on the walls of the test vessels. Additionally, no interferences of the ten different sediment and soil sample matrices were observed in the chromatograms under the specified experimental conditions, which gives the method sufficient selectivity. The characteristic mass spectra of the blank sediment samples in 0.01 M CaCl_2_ and 2 mg/L CEF, MEM and PRAZ in 0.01 M CaCl_2_ are shown in Figure 1.

It can be seen from Figure 1 that sediment sample 1 (Glina) did not contain any of the investigated pharmaceuticals (CEF, MEM and PRAZ). The absence of the characteristic mass spectra of the mentioned pharmaceuticals (specific precursor ion/production transitions for each of the investigated pharmaceuticals) is sufficient evidence for this. Identical results were obtained for other sediment and soil samples in support of the above results, i.e., the fact that the soil and sediment samples used do not contain pharmaceuticals, in previously publications [13,40]

The linear sorption isotherms of all three pharmaceuticals tested are shown in Supplementary Information, Figure S2. Table 1, Table 2 and Table 3 show the sorption coefficients and all other parameters derived from three different models of sorption isotherms (linear, Freundlich, and Dubinin–Radushkevich) for each soil and sediment tested.

From the obtained regression coefficients *R*^2^, it can be concluded that only the linear isotherm describes the sorption process with a value of *R*^2^ > 0.989 in the case of all tested pharmaceuticals, while the regression coefficients for the Freundlich isotherm generally range between 0.738–0.993. It is also evident from the presented tables that the Dubinin–Radushkevich sorption model does not describe the situation very well for all three tested pharmaceuticals (*R*^2^ ranges from 0.482–0.999), but this was not achieved with any other applied model for all pharmaceuticals tested simultaneously. The Dubinin–Radushkevich model, based on the value of free sorption energy *E* (kJ/mol), was chosen only because, unlike all other models, it can inform the reader about the sorption mechanism operating between the studied pharmaceuticals and the soil/sediment samples [9]. The Dubinin–Radushkevich isotherm shows the worst agreement with the experimental data, and its regression coefficients are generally in the range of 0.671–0.999, although there are data whose *R*^2^ is below the indicated range in the case of MEM. On the basis of the calculated *E* values shown in Table 1, Table 2 and Table 3, sorption between all tested pharmaceuticals and sorbents was characterized as a physical process based on weak van der Waals forces.

Based on the values obtained for MEM (*K*_d_ ranging from 0.75–38.51 mL/g, *K*_F_ ranging from 0.91–66.07 ((μg/g)(mL/μg)^1/*n*^), and the *q*_m_ values referring to the amount of maximum adsorbed substance), it is clear that MEM binds less to the tested sediments and soils compared to other tested pharmaceuticals. Comparing the *K*_d_ values in general for all three tested pharmaceuticals, it is observed that the highest *K*_d_ values were obtained for CEF (*K*_d_ = 10.69–972.73 mL/g), although the highest *K*_d_ values were still obtained between soil 1 and PRAZ. PRAZ showed better mobility in soil and sediment samples with lower *K*_d_ values compared to CEF (*K*_d_ = 0.27–69.70 mL/g) and bound less to the sediment and soil samples tested than the other pharmaceuticals compared to CEF, although it bound better than MEM.

From the values presented in Table 1, Table 2 and Table 3, it can be seen that for CEF in the experiments with the addition of 100 mg/L HA, generally all values of *n* are less than 1 (0.16–0.64). This indicates lower sorption for the tested soils and sediments compared to other pharmaceutical products tested, and also indicates higher heterogeneity of the sorbate surface. The *n*-values obtained for MEM are higher than 1 for seven samples, indicating higher sorption intensity. PRAZ showed constant sorption affinity over the entire concentration range, with *n*-values close to 1 for almost all soil and sediment samples used. Exceptions were observed in the cases of sediment 3 with 0 mg/L HA and sediment 4 with 100 mg/L HA (*n* = 2.584), sediment 4 (*n* = 1.736), and sediment 3 (*n* = 2.070), supporting the fact that more and more PRAZ molecules are sorbed near the soil/sediment and that the previously sorbed molecules enhance further sorption by modifying the mentioned surfaces. For CEF, *n* > 1 is only in the case for soil 1 (*n* = 1.5198), soil 2 (*n* = 1.8560), sediment 4 (*n* = 1.370) and sediment 3 (*n* = 1.1350), while MEM reaches all values for the soil/sediment samples studied (*n* = 1.5868–3.2020). In general, howeverm the *K*_d_ values indicate a low binding affinity of MEM. 

### 3.3. Influence of Organic Matter and Composition of Soil/Sediment Samples

Soil organic matter is a complex, heterogeneous material [16] called humus. Humus consists of humic and fulvic acids and humin. The organic component of soil or sediment is one of the most influential parameters affecting the sorption of pharmaceuticals, which is confirmed by numerous works from the literature [21,24,41,42,43]. The effect of soil organic components on pharmaceutical mobility or sorption is twofold. This notion is supported by available research [44], where increased sorption was observed at a lower concentration of OM (1 mg/L), in contrast to a higher concentration of humic acid (10 mg/L), which resulted in an opposite effect. On the other hand, studies by Ling et al. [45] show that the binding of OM to sediment particles increases the organic carbon content and changes the surface morphology, which directly leads to an increase in adsorption sites on the sediment surface, i.e., increases the adsorption of the tested component (oxytetracycline). This is because organic matter can form complexes with relatively polar compounds or absorb hydrophobic organic contaminants, apparently increasing water solubility and possibly decreasing sorption. In addition, the presence of organic matter can promote adsorption of pharmaceuticals, and the higher the concentration of OM is, the more pronounced this effect becomes [41]. 

**Figure 2:**
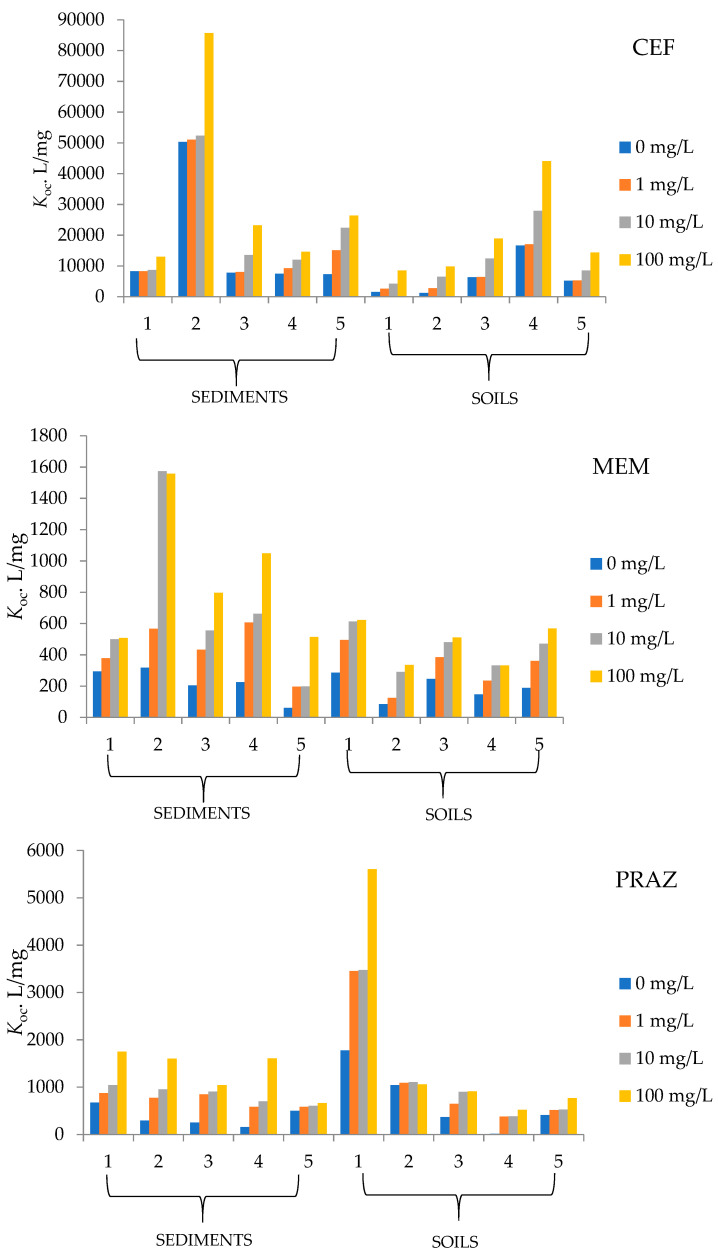
Soil and sediment sorption coefficients for tested pharmaceuticals.

Since the pharmaceuticals used in this paper are relatively unexplored in terms of their behavior in solid environmental samples (soil, sediment), the question arises as to how they behave in the presence of different amounts of organic matter. To determine whether and how OM affects the sorption of CEF, MEM, and PRAZ to the soil and sediment samples studied, experiments were conducted with the addition of humic acids. Just as the partition coefficient (*K*_d_) increases with the amount of organic matter, the normalized distribution constant of organic carbon content (*K*_oc_) also increases with an increasing amount of organic carbon. The results are shown in Figure 2.

Sediment 5 and soil 2 in the case of MEM have the lowest partition coefficients at 0 and 100 mg/L humic acids, indicating that these samples have the lowest affinity for sorption. On the other hand, sediment 2 and soil 5 have the highest affinity among the studied soils and sediments, as their partition coefficients are highest at 100 mg/L humic acids, being 13.10 and 38.51 mL/g, respectively. The *K*_oc_ values in the case of MEM sorption range from 60.81 to 1557.9 mL/g.

Considering the other tested pharmaceuticals, we find that *K*_d_ reaches the highest values for CEF sorption rather than for MEM and PRAZ. The value is highest for sediment 2 (720.4 mg/L) and soil 5 (972.7 mg/L) and in the case of MEM. From the physico-chemical properties of soil 5, there is a very high humus content of 11.59%, the highest of all sediments and soils studied, as well as the highest conductivity (144.30 µS/cm) and a TDS value of 92.40 mg/L. The physico-chemical properties reported for soil 5 suggest that the high value of the above factors has a great influence on the sorption capacity of soil 5. Sediment 4 and soil 2 have the lowest values of partition coefficients of 100.7 and 92.25 mg/L, respectively. In addition to soil 5 and sediment 2, soils 3 and 4 also have large sorption capacities. Since large amounts of partition coefficients were recorded, CEF resulted in large *K*_oc_ values, ranging from 1213.0 to 85,655.4 mL/g.

An increase in humic acid concentration results in greater sorption to the solid phase and with respect to PRAZ. This is reflected in a series of increases in the partition coefficients for all five soils studied, as well as for the sediment samples. The ratio of the equilibrium concentration of sorbed substance and residue in solution is highest for soil 1 with 116.42 mL/g and for sediment 1 with 31.45 mL/g. On the other hand, the sorption of PRAZ under organic influence is weakest in soil 4 because the distribution between solid and liquid phases is 9.609 mL/g at a HA concentration of 100 mg/L. In the calculation of *K*_oc_, values between 15.3 and 5606.37 mL/g were obtained depending on the organic matter content.

It is very important to point out that increasing the concentration of HA also affects other physicochemical properties of the soil/sediment. For example, there is a non-negligible effect on the pH of soil and sediment samples, which can easily shift towards more acidic values with an increase in the concentration of HA [24]. It is important to note that this change is not the same for all sediment and soil samples studied, as some soils and sediments are resistant to pH changes due to their high buffering capacity, which is due to the presence of various organic acids and other soil/sediment components that can form neutral salts. 

The study of the influence of organic matter on sorption is certainly of greater importance. Humic acids should not be considered as the only organic component that can influence sorption and as such can be found in the soil/sediment. It is convenient to observe because it is a natural component of soil and sediment and is very readily available for the experimental part since it can be extracted and then easily tested. Humic acids here can stand for any organic component/substance that occurs in soil/sediment and thus affects the behavior of pharmaceuticals in the environment. For these three tested pharmaceuticals (CEF, MEM and PRAZ), it is observed that as the concentration of organic matter (in the form of humic acids) increases, their sorption in soil and sediment increases, which significantly affects their mobility in the environment. In this respect, CEF is the most immobile in the environment, as soils/sediments rich in organic matter are less hazardous to water than to aquatic organisms. At the same time, soils/sediments rich in organic matter are not able to bind PRAZ, especially MEM, which poses a potential hazard in water. 

Since sorption depends not only on the physicochemical properties of the test substances, but also on the physicochemical properties of the studied soils and sediments, a correlation was performed for all studied pharmaceuticals to determine how changes in humic acid concentration affect the physicochemical parameters of the soils/sediments.

Based on the Pearson coefficient [7] from Table 4, it can be seen that the different physicochemical parameters do not have the same influence on the sorption of all three tested pharmaceuticals. In the case of CEF, there is a weak correlation between *K*_d_ and the texture of the soil/sediment samples (coarse sand, silt, and fine sand content), while other physicochemical parameters indicate that there is no significant linear correlation, as the *r* values generally range from 0 to ±0.25. With increasing humic acid concentration, the association increases in the case of silt and CEC content of Zn, Cu and Mn, although all this is not significant. However, in practice, it is known that the significance of the correlation coefficient is evaluated at the significance level. So, based on *p* < 0.05, we conclude that Pearson’s linear correlation coefficient is statistically significant in several cases, i.e., we can say that there is no statistically significant correlation between *K*_d_ CEF values and coarse sand, OM, CaCO_3_, CEC content and Cu content, as *p* < 0.05. For other parameters, we also cannot claim this with statistical certainty, since *p* is slightly higher than 0.05, and so a larger number of measurements should be performed. However, in the case of memantine, the situation is somewhat different. Based on the Pearson coefficients presented in Table 4, a weak correlation is observed between *K*_d_ for MEM and coarse sand, fine sand, CEC and CaCO_3_, this correlation being negative in the case of coarse sand and CaCO_3_ content. A moderate coupling is observed for pH, while the coupling is excellent in the case of EC or total dissolved salts (TDS). An increase in humic acid concentration leads to a decrease in the Pearson coefficient in almost all cases (the only exceptions are clay, OM and the percentage of Mn, Cu and Fe). In the case of EC and TDS, where the initial value of the Pearson coefficient (γ (HA) = 0 mg/L) indicates an excellent association with *K*_d_ for MEM, an increase in humic acid concentration (γ (HA) = 100 mg/L) also resulted in a decrease in the association to a nonsignificant level. The evaluation of the significance level of the calculated correlation coefficients showed that there is no correlation between the studied variables (*r* < ±0.25) for clay and the proportions of Zn, Cu, Fe and Mn, as the correlation coefficient is significant (*p* < 0.03). On the basis of *p* < 0.05, it can be claimed that the correlation between *K*_d_ and OM increases significantly (on the basis of the value of Pearson’s coefficient, the transition from non-significant to weak correlation was observed), while the situation is reversed for coarse sand and CE. In PRAZ, a strong correlation (*r* = 0.50–0.75) was observed between *K*_d_ and the content of clay, which changed slightly with the increasing concentration of humic acids (*p* < 0.05). The content of Fe and Mn in soils/sediments significantly affected the *K*_d_ value of PRAZ (*p* < 0.05). For other physicochemical parameters, no correlation was found between *K*_d_ values with or without an increase in humic acid concentration. 

From the correlation analysis performed for all three tested pharmaceuticals (CEF, MEM and PRAZ), it can be concluded that pH does not change significantly with increasing humic acid concentration. This is supported by the fact that all tested soils and sediments have a high buffering capacity and resist pH changes due to the formation of neutral salts between the metal ions and the humic acids or organic acids present, which is confirmed by the above data. 

The experimentally studied dependence of the organic component of the soil/sediment on the sorption coefficient *K*_d_ of the tested pharmaceuticals and the performed correlation analysis confirm again that sorption is a complex process that depends not only on the physicochemical properties of the tested pollutants, but also on the physicochemical properties of the soil/sediment.

### 3.4. Sorption Kinetics

The kinetic study of the pharmaceutical sorption process was performed at three concentration levels to cover the entire range used in other experiments. The sorption results were interpreted using two kinetic models—Lagergren’s (pseudo-first order) and Ho´s (pseudo-second order) (Equations (7) and (8)) [46,47].

(7) $$\ln\left(q_{e}-q_{t}\right)=lnq_{e}-k_{1}t$$

(8) $$\frac{t}{q_{t}}=\frac{1}{k_{2}q_{e}^{2}}+\frac{t}{q_{e}}$$

where *q*_t_ and *q*_e_ represent the amount of sorbed analyte at time t and at equilibrium (μg/g), *k*_1_ is the constant rate pseudo-first-order value (min^−1^), and *k*_2_ is the constant rate of kinetic model pseudo-second-order value (g/μg min). The obtained experimental *q*_e_ values were closer to the values calculated with the pseudo-second-order kinetics, which means that mentioned model is more suitable for describing the sorption process (which is also confirmed by *R*^2^ to 1).

Table 5 clearly shows that the pseudo-second-order kinetic model describes the sorption kinetics much better than the pseudo-first-order model (*R*^2^ in the range of 0.9996–1.0000) for all the pharmaceuticals tested. Using this model, the maximum concentrations (*q*_e_) of the sorbed tested components that are in equilibrium were calculated for the environmental samples tested. These *q*_e_ values range from 16.668–19.484 μg/g for all tested pharmaceuticals (0.796–19.484 μg/g for CEF; 0.989–16.668 μg/g for MEM; 0.685–18.649 μg/g for PRAZ).

### 3.5. Sorption Thermodynamics

The obtained *K*_d_ values are inserted into Equation (9) to describe the thermodynamics of the process by calculating the Gibbs free energy [48].

(9) $$\Delta G^{{}^{\circ}}=-RTlnK_{d}$$

As shown in Table 6, cefdinir, memantine, and praziquantel were spontaneously sorbed to the soil and sediment samples studied by physisorption due to weak Van der Waals forces, which is confirmed with negative $\Delta G^{{}^{\circ}}$ values ranging from −17.046 to 0.05 kJ/mol [43,49]. These statements are also consistent with the energy calculated from Dubinin–Radushkevich isotherms (see Section 3.2).

## 4. Conclusions

For the first time, to the author’s knowledge, the influence of organic matter has been studied as an important component of solid environmental samples such as soils and sediments through the sorption of three different pharmaceuticals. *R^2^* values close to one for a linear isotherm showed good agreement between the experimental data and the model, whose considerations can also be applied to the sorption system studied. The addition of humic acids in the sorption process between pharmaceutical and sorbent promoted the sorption affinity of CEF, MEM and PRAZ with soils and sediments. Depending on the sorbent used and its physicochemical properties, a greater or lesser change in the partition coefficient was observed. In general, a higher content of organic matter in the solution promoted binding between the tested pharmaceuticals and all soil and sediment samples. A high sorption affinity can be observed for CEF, with the highest *K*_d_ values ranging from 31.84 to 972.7 mL/g, while PRAZ and MEM generally show a high sorption behavior of CEF, and as PRAZ and MEM are expected to be more exposed in the environment, which was confirmed by low *K*_d_, *K*_F_ and *q*_m_ values. MEM clearly stands out as the most mobile component among the tested pharmaceuticals, tending to the aquatic environment with a *K*_d_ value below 38.5 mL/g. Thus, its fate in the environment does not depend on the sorption process, but rather on other biotic processes such as photolysis and hydrolysis. The good agreement between the experimental results and Freundlich isotherm model describes sorption for all tested pharmaceuticals as a complex process occurring in both one and multiple layers of the sorbents used. The pseudo-second-order kinetic model showed the best agreement with the obtained results with an R^2^ greater than 0.99. The thermodynamics were spontaneous, with CEF, MEM and PRAZ being sorbed by physisorption to the soil and sediment samples. The energy values determined using the Dubinin–Raduschkevich isotherm <8 kJ/mol confirmed the physical nature of the sorption.

Finally, this research confirmed the complexity of sorption, which is influenced by various process parameters. By focusing on the characterization of the sorbent and the pharmaceuticals, researchers can promote or reduce the mobility of contaminants in the environment. This and all similar research contribute to a better understanding of the various theoretical aspects of contaminant fate and behavior in different environments.

## Figures and Tables

**Table 1:** The linear, Freundlich, and Dubinin–Radushkevich isotherm parameters for CEF.

Isotherm	HA	0 mg/L					1 mg/L					10 mg/L					100 mg/L				
	Sediment/ Soil	1	2	3	4	5	1	2	3	4	5	1	2	3	4	5	1	2	3	4	5
		1	2	3	4	5	1	2	3	4	5	1	2	3	4	5	1	2	3	4	5
Linear	*K*_d_	143.2	393.7	113.0	47.30	117.3	143.3	399.7	116.6	58.55	242.4	151.9	413.0	197.7	76.40	341.1	233.1	720.4	351.0	100.8	438.5
		31.84	10.69	144.5	297.6	350.3	52.68	24.39	147.2	304.2	355.4	84.82	57.53	284.4	499.9	573.4	176.7	92.26	444.9	813.1	972.7
	*R* ^2^	0.996	0.998	0.995	0.996	0.991	0.998	0.998	0.996	0.996	0.994	0.993	0.993	0.998	0.999	0.932	0.998	0.992	0.992	0.999	0.995
		0.992	0.990	0.998	0.991	0.998	0.990	0.991	0.993	0.992	0.995	0.993	0.994	0.993	0.996	0.990	0.992	0.992	0.989	0.992	0.990
Freundlich	*K*_F_	202.3	3342.0	92.26	34.36	169.0	182.4	261.8	54.58	34.67	105.4	82.04	261.8	85.90	31.33	181.6	6854.9	4.44 × 10^7^	4.92 × 10^4^	353.2	3.54 × 10^5^
		23.61	9.89	264.2	2741.6	2944.4	28.45	27.54	208.9	335.7	234.4	41.21	32.58	107.6	295.8	386.4	1798.9	223.36	2.61 × 10^5^	2.35 × 10^8^	4.62 × 10^9^
	*n*	0.87	0.57	1.14	1.37	0.90	0.91	1.15	1.58	1.59	1.48	1.27	1.20	1.40	1.75	1.35	0.39	0.20	0.33	0.57	0.27
		1.52	1.86	0.79	0.55	0.57	1.71	1.03	0.90	0.97	1.11	1.59	1.54	1.44	1.18	1.09	0.47	0.64	0.28	0.18	0.16
	*R* ^2^	0.938	0.945	0.953	0.976	0.941	0.975	0.994	0.992	0.984	0.987	0.941	0.987	0.976	0.948	0.975	0.932	0.822	0.862	0.956	0.894
		0.952	0.979	0.987	0.923	0.931	0.962	0.978	0.987	0.993	0.986	0.965	0.962	0.953	0.982	0.985	0.874	0.890	0.808	0.835	0.839
D-R	*q*_m_	40.38	168.17	26.00	14.72	40.06	36.14	37.85	16.99	14.52	25.73	17.99	40.33	19.62	11.46	35.02	294.4	3.07 × 10^3^	827.1	63.19	2.27 × 10^3^
		12.91	6.62	45.31	171.2	165.1	11.09	15.54	40.19	43.10	33.03	14.41	12.06	23.85	41.34	43.84	151.3	47.93	2.13 × 10^3^	6.71 × 10^4^	3.24 × 10^5^
	*β*	0.030	0.040	0.022	0.020	0.030	0.027	0.015	0.012	0.015	0.012	0.015	0.014	0.013	0.011	0.013	0.079	0.140	0.088	0.061	0.11
		0.020	0.017	0.033	0.046	0.042	0.012	0.040	0.027	0.020	0.016	0.013	0.014	0.012	0.014	0.015	0.068	0.054	0.100	0.15	0.17
	*E*	4.10	3.55	4.81	5.01	4.06	4.28	5.77	6.45	5.87	6.48	5.79	5.93	6.27	6.84	6.30	2.52	1.89	2.38	2.87	2.13
		5.01	5.44	3.88	3.30	3.45	6.51	3.54	4.27	5.04	5.63	6.25	5.95	6.48	5.98	5.79	2.72	3.04	2.24	1.83	1.71
	*R* ^2^	0.947	0.962	0.951	0.934	0.970	0.975	0.978	0.937	0.939	0.978	0.849	0.984	0.911	0.828	0.979	0.959	0.839	0.882	0.989	0.911
		0.928	0.835	0.998	0.955	0.953	0.833	0.990	0.999	0.976	0.948	0.894	0.848	0.921	0.974	0.965	0.901	0.904	0.830	0.845	0.849

*K*_d_—mL/g; *K*_F_—(μg/g)(mL/μg)^1/*n*^; *q*_m_—μg/g; *β—*mol^2^/kJ^2^; *E*—kJ/mol. Blue numbers correspond to sediment samples; black number correspond to soil samples (see the list of used sediment and soil samples in Section 2.2).

**Table 2:** The linear, Freundlich, and Dubinin–Radushkevich isotherm parameters for MEM.

Isotherm	HA	0 mg/L					1 mg/L					10 mg/L					100 mg/L				
	Sediment/ Soil	1	2	3	4	5	1	2	3	4	5	1	2	3	4	5	1	2	3	4	5
		1	2	3	4	5	1	2	3	4	5	1	2	3	4	5	1	2	3	4	5
Linear	*K*_d_	5.11	2.49	2.97	1.43	0.98	6.57	4.44	6.30	3.84	3.15	8.71	12.42	8.11	4.23	3.20	9.11	13.10	12.05	7.24	8.54
		5.76	0.75	5.64	2.62	12.66	9.99	1.10	8.81	4.20	24.29	12.42	2.58	11.02	5.95	31.71	12.91	3.15	12.00	6.13	38.51
	*R* ^2^	0.993	0.991	0.991	0.992	0.993	0.994	0.992	0.996	0.992	0.990	0.993	0.994	0.990	0.990	0.995	0.993	0.996	0.998	0.990	0.998
		0.993	0.994	0.993	0.991	0.991	0.997	0.993	0.997	0.991	0.996	0.993	0.992	0.991	0.991	0.990	0.994	0.994	0.993	0.990	0.991
Freundlich	*K*_F_	4.32	2.68	3.13	1.64	1.03	6.50	4.21	6.34	3.34	2.67	7.10	9.79	7.52	4.51	3.42	12.68	6.93	12.59	5.56	8.07
		4.85	0.91	5.01	2.82	7.75	8.34	1.47	7.41	4.03	21.23	9.80	3.29	9.68	6.37	13.09	9.86	2.99	9.75	5.09	66.07
	*n*	1.81	1.99	2.05	1.91	1.59	1.76	1.54	1.48	1.53	1.09	2.82	2.12	2.22	1.97	1.67	0.44	2.40	0.78	2.86	1.22
		1.95	1.86	1.75	1.97	3.21	1.43	1.88	1.84	1.68	1.34	2.12	1.86	2.16	2.04	2.76	1.51	1.19	1.46	1.32	0.595
	*R* ^2^	0.865	0.888	0.889	0.918	0.893	0.970	0.949	0.982	0.881	0.876	0.915	0.949	0.950	0.981	0.930	0.917	0.746	0.989	0.842	0.991
		0.866	0.890	0.893	0.882	0.867	0.949	0.952	0.938	0.906	0.994	0.949	0.976	0.986	0.977	0.857	0.950	0.976	0.950	0.904	0.988
D-R	*q*_m_	3.25	2.34	2.68	1.52	1.01	4.83	3.45	4.91	2.70	2.27	4.88	6.11	5.14	4.04	2.85	12.58	4.35	9.28	3.96	6.07
		3.61	1.15	3.81	2.41	5.14	5.56	1.42	5.02	3.18	9.90	6.11	2.99	6.45	4.76	6.77	6.44	2.82	6.15	3.74	25.16
	*β*	0.020	0.023	0.021	0.029	0.037	0.021	0.030	0.030	0.029	0.050	0.0077	0.012	0.012	0.021	0.027	0.17	0.0086	0.0733	0.0077	0.039
		0.018	0.031	0.022	0.022	0.0055	0.027	0.031	0.017	0.025	0.020	0.012	0.027	0.013	0.017	0.0055	0.023	0.051	0.024	0.033	0.078
	*E*	4.98	4.65	4.86	4.18	3.67	4.85	4.08	4.10	4.12	3.16	8.06	6.48	6.40	4.88	4.28	1.73	7.62	2.61	8.06	3.57
		5.35	4.00	4.76	4.74	9.53	4.34	4.04	5.39	4.50	4.98	6.48	4.27	6.25	5.46	9.53	4.69	3.13	4.55	3.89	2.53
	*R* ^2^	0.584	0.637	0.648	0.648	0.602	0.782	0.734	0.822	0.604	0.618	0.685	0.750	0.732	0.815	0.686	0.970	0.482	0.947	0.575	0.869
		0.604	0.595	0.659	0.618	0.642	0.753	0.717	0.714	0.646	0.882	0.750	0.833	0.824	0.790	0.646	0.767	0.811	0.765	0.654	0.990

*K*_d_—mL/g; *K*_F_—(μg/g)(mL/μg)^1/*n*^; *q*_m_—μg/g; *β—*mol^2^/kJ^2^; *E*—kJ/mol. Blue numbers correspond to sediment samples; black number correspond to soil samples (see the list of used sediment and soil samples in Section 2.2).

**Table 3:** The linear, Freundlich, and Dubinin–Radushkevich isotherm parameters for PRAZ.

Isotherm	HA	0 mg/L					1 mg/L					10 mg/L					100 mg/L				
	Sediment/ Soil	1	2	3	4	5	1	2	3	4	5	1	2	3	4	5	1	2	3	4	5
		1	2	3	4	5	1	2	3	4	5	1	2	3	4	5	1	2	3	4	5
Linear	*K*_d_	11.73	2.29	3.68	1.01	8.05	15.23	6.06	12.32	3.69	9.39	18.23	7.52	13.21	4.47	9.72	31.46	13.45	15.76	11.09	11.03
		35.84	9.19	8.41	0.27	27.75	69.70	9.62	14.81	6.80	34.58	70.29	9.79	20.61	6.90	35.41	116.42	9.96	21.43	9.61	52.12
	*R* ^2^	0.993	0.998	0.994	0.991	0.997	0.993	0.992	0.992	0.993	0.994	0.990	0.992	0.995	0.995	0.993	0.997	0.995	0.992	0.994	0.997
		0.992	0.996	0.986	0.992	0.997	0.990	0.993	0.992	0.995	0.989	0.994	0.994	0.999	0.991	0.998	0.996	0.996	0.995	0.991	0.996
Freundlich	*K*_F_	17.74	2.22	4.09	1.30	8.24	16.56	6.89	13.09	3.76	8.71	17.46	7.87	14.06	4.95	10.42	36.81	14.93	17.50	11.64	11.07
		38.64	11.17	14.06	0.49	39.45	115.08	11.38	17.22	7.03	43.65	101.16	12.02	23.28	7.64	40.00	201.84	9.68	23.99	10.02	63.53
	*n*	0.52	0.60	2.07	1.74	1.00	1.10	1.24	1.17	1.52	0.99	0.97	0.94	1.00	1.01	0.60	0.93	0.96	0.99	1.00	0.97
		0.92	0.53	0.52	2.58	0.69	0.80	0.91	0.99	1.25	0.92	0.95	0.96	0.99	0.98	0.98	0.78	0.98	0.97	1.29	0.92
	*R* ^2^	0.851	0.925	0.926	0.943	0.981	0.978	0.992	0.981	0.958	0.964	0.972	0.954	0.980	0.976	0.988	0.982	0.981	0.970	0.984	0.990
		0.947	0.932	0.738	0.935	0.935	0.920	0.907	0.956	0.988	0.938	0.946	0.957	0.988	0.975	0.982	0.947	0.992	0.978	0.954	0.967
D-R	*q*_m_	16.92	3.11	3.25	1.27	6.98	11.48	6.01	9.70	3.27	6.46	11.05	6.98	10.10	5.01	8.61	18.00	10.65	11.70	8.57	8.29
		17.95	10.64	1.06	0.50	20.40	35.99	5.18	12.51	5.81	21.62	31.42	10.58	13.91	6.83	18.40	41.87	7.43	14.43	8.10	24.10
	*β*	0.14	0.14	0.018	0.035	0.058	0.042	0.044	0.041	0.034	0.053	0.048	0.065	0.051	0.066	0.11	0.043	0.053	0.048	0.053	0.055
		0.043	0.14	0.15	0.025	0.072	0.043	0.005	0.052	0.042	0.044	0.039	0.12	0.055	0.061	0.041	0.037	0.055	0.047	0.038	0.038
	*E*	1.90	1.91	7.47	3.79	2.94	3.43	3.36	3.48	3.86	3.08	3.23	2.78	3.14	2.75	2.15	3.39	3.06	3.23	3.09	3.02
		3.41	1.92	2.56	4.48	2.64	3.41	10.43	3.10	3.47	3.37	3.60	2.05	3.02	2.87	3.49	3.66	3.01	3.28	3.62	3.63
	*R* ^2^	0.937	0.972	0.671	0.718	0.938	0.979	0.947	0.970	0.772	0.850	0.936	0.926	0.959	0.962	0.954	0.990	0.967	0.959	0.933	0.937
		0.941	0.963	0.881	0.675	0.953	0.958	0.923	0.980	0.904	0.976	0.971	0.973	0.981	0.953	0.984	0.960	0.924	0.987	0.935	0.986

*K*_d_—mL/g; *K*_F_—(μg/g)(mL/μg)^1/*n*^; *q*_m_—μg/g; *β—*mol^2^/kJ^2^; *E*—kJ/mol. Blue numbers correspond to sediment samples; black number correspond to soil samples (see the list of used sediment and soil samples in Section 2.2).

**Table 4:** Change in Pearson’s correlation coefficients between soil/sediment physicochemical properties and *K*_d_ value of studied pharmaceuticals with change in OM concentration (from 0 to 100 mg/L humic acid).

Parameter	CEF	MEM	PRAZ
Coarse sand	−0.490 to −0.459	−0.408 to −0.309	0.209 to 0.109
Clay	−0.278 to −0.233	−0.127 to −0.162	0.665 to 0.640
Silt	0.393 to 0.454	0.223 to −0.003	−0.371 to −0.288
Fine sand	0.490 to 0.458	0.408 to 0.310	−0.209 to −0.109
pH	0.125 to −0.023	0.616 to 0.377	0.228 to 0.382
EC	0.198 to 0.100	0.753 to 0.370	−0.274 to 0.133
TDS *	0.198 to 0.100	0.753 to 0.370	−0.274 to 0.133
CEC	−0.184 to −0.213	0.413 to −0.138	−0.102 to 0.014
OM	−0.257 to −0.258	−0.035 to −0.279	−0.015 to −0.111
CaCO_3_	−0.182 to −0.189	−0.404 to −0.227	0.404 to −0.007
Zn	−0.077 to −0.117	−0.023 to −0.031	−0.179 to −0.232
Cu	−0.071 to −0.085	−0.005 to −0.063	−0.327 to −0.357
Fe	0.210 to 0.184	0.093 to 0.175	−0.556 to −0.430
Mn	−0.007 to −0.062	0.232 to 0.029	−0.570 to −0.420

TDS * = Total Dissolved Solids.

**Table 5:** Sorption kinetic parameters of pharmaceuticals for soil 5.

	Initial Concentration, mg/L	*q*_e,exp_, μg/g	Pseudo-First-Order			Pseudo-Second-Order		
			*q*_e,calc_, μg/g	*k*_1_, min^−1^	*R* ^2^	*q*_e,calc_, μg/g	*k*_2_, g/μg min	*R* ^2^
CEF	2.0	19.484	2.1918	1.15·10^−3^	0.8065	19.5313	0.011	1.0000
	0.5	4.795	0.8484	1.15·10^−3^	0.9029	4.8193	0.024	0.9999
	0.1	0.796	0.2349	1.2·10^−4^	0.3672	3.9078	0.039	1.0000
MEM	2.0	16.668	5.7438	4.606·10^−4^	0.7940	16.7504	0.00640	0.9999
	0.5	4.772	0.8385	1.152·10^−3^	0.7305	4.7847	0.02906	1.0000
	0.1	0.989	0.0633	1.382·10^−2^	0.7346	0.9896	0.38227	1.0000
PRAZ	2.0	18.649	5.507	1.152·10^−3^	0.9064	18.832	0.03311	1.0000
	0.5	4.011	1.649	4.606·10^−4^	0.8256	4.036	0.02157	0.9997
	0.1	0.685	0.377	1.612·10^−4^	0.4106	0.686	0.34342	0.9996

**Table 6:** Standard Gibbs free energy calculated for different concentration of HA.

	ΔG°, kJ/mol			
	0 mg/L HA	1 mg/L HA	10 mg/L HA	100 mg/L HA
**CEF**				
sediment 1	−12.30	−12.30	−12.45	−13.51
sediment 2	−14.80	−14.84	−14.92	−16.30
sediment 3	−11.71	−11.79	−13.10	−14.52
sediment 4	−9.55	−10.08	−10.74	−11.43
sediment 5	−11.81	−13.60	−14.59	−15.07
soil 1	−8.57	−9.82	−11.00	−12.82
soil 2	−5.87	−7.91	−10.04	−11.21
soil 3	−12.32	−12.37	−14.00	−15.11
soil 4	−14.11	−14.17	−15.40	−16.60
soil 5	−14.52	−14.55	−15.74	−17.05
**MEM**				
sediment 1	−4.04	−4.66	−5.36	−5.47
sediment 2	−2.26	−3.69	−6.24	−6.37
sediment 3	−2.70	−4.56	−5.19	−6.16
sediment 4	−0.89	−3.33	−3.57	−4.90
sediment 5	0.05	−2.84	−2.88	−5.31
soil 1	−4.34	−5.70	−6.24	−6.34
soil 2	0.75	−0.24	−2.35	−2.84
soil 3	−4.29	−5.39	−5.95	−6.16
soil 4	−2.39	−3.56	−4.42	−4.49
soil 5	−6.29	−7.90	−8.56	−9.05
**PRAZ**				
sediment 1	−6.10	−6.75	−7.19	−8.54
sediment 2	−2.06	−4.46	−5.00	−6.44
sediment 3	−3.23	−6.22	−6.39	−6.83
sediment 4	−0.02	−3.23	−3.71	−5.96
sediment 5	−5.17	−5.55	−5.63	−5.95
soil 1	−8.87	−10.52	−10.54	−11.79
soil 2	−5.50	−5.61	−5.65	−5.69
soil 3	−5.28	−6.68	−7.50	−7.59
soil 4	3.24	−4.75	−4.79	−5.61
soil 5	−8.23	−8.78	−8.84	−9.80

## Data Availability

The data presented in this study are available upon request from the corresponding author.

## Supplementary Materials

The following supporting information can be downloaded at: https://www.mdpi.com/article/10.3390/molecules27228008/s1, Figure S1: Kinetics for pharmaceuticals on the investigated soil and sediment samples: (a) CEF- soil 1 (b) CEF- sediment 1 (c) MEM- soil 5 (d) MEM- sediment 1 (e) PRAZ- soil 5 and (f) PRAZ- sediment 2. T = 25 °C; Figure S2: Linear sorption isotherms of CEF, MEM and PRAZ in five sediment samples and in five soil samples at 25 °C; Table S1: Selected pharmaceuticals, their structures and physico-chemical properties [33]; Table S2: Physico-chemical characterization of sediment and soil samples.

## Sample Availability

Not available.
