# Supplementary material for: Influence of Organic Matter on the Sorption of Cefdinir, Memantine and Praziquantel on Different Soil and Sediment Samples
Source: Molecules. 2022 Nov 18;27(22):8008. doi: 10.3390/molecules27228008 (PMC9696219; doi:10.3390/molecules27228008)
Supplement: Supplementary file 1 [file molecules-27-08008-s001.zip › molecules-2019189-supplementary.pdf]

Supplementary Information

Influence of Organic Matter on the Sorption of Cefdinir, Memantine and Praziquantel on Different Soil and Sediment Samples

Dragana Mutavdžić Pavlović [1,*], Kristina Tolić Čop [1], Helena Prskalo [1] and Mislav Runje [2]

[1] Department of Analytical Chemistry, Faculty of Chemical Engineering and Technology, University of Zagreb, 10000 Zagreb, Croatia
[2] Pliva Croatia TAPI R&D, 10000 Zagreb, Croatia

List of figures:

Figure S1. Kinetics for pharmaceuticals on the investigated soil and sediment samples: a) CEF- soil 1 b) CEF- sediment 1
c) MEM- soil 5 d) MEM- sediment 1 e) PRAZ- soil 5 and f) PRAZ- sediment 2. T = 25 °C.

Figure S2. Linear sorption isotherms of CEF, MEM and PRAZ in five sediment samples and in five soil samples at 25 °C.

List of tables:

Table S1. Selected pharmaceuticals, their structures and physico-chemical properties [33]

Table S2. Physico-chemical characterization of sediment and soil samples

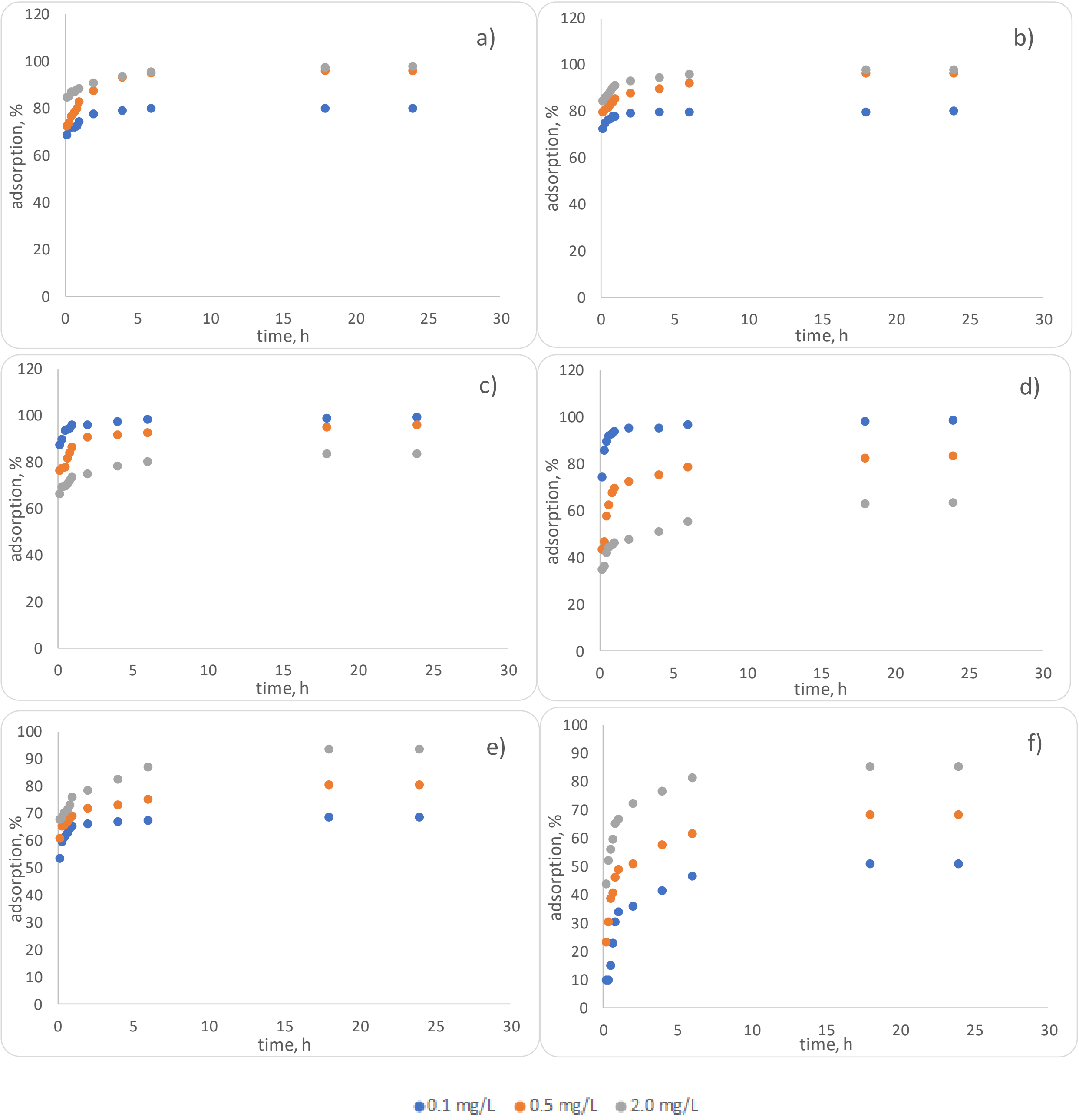

Figure S1. Kinetics for pharmaceuticals on the investigated soil and sediment samples: (**a**) CEF- soil 1 (**b**) CEF- sediment 1 (**c**) MEM- soil 5 (**d**) MEM- sediment 1 (**e**) PRAZ- soil 5 and (**f**) PRAZ- sediment 2. T = 25 °C.

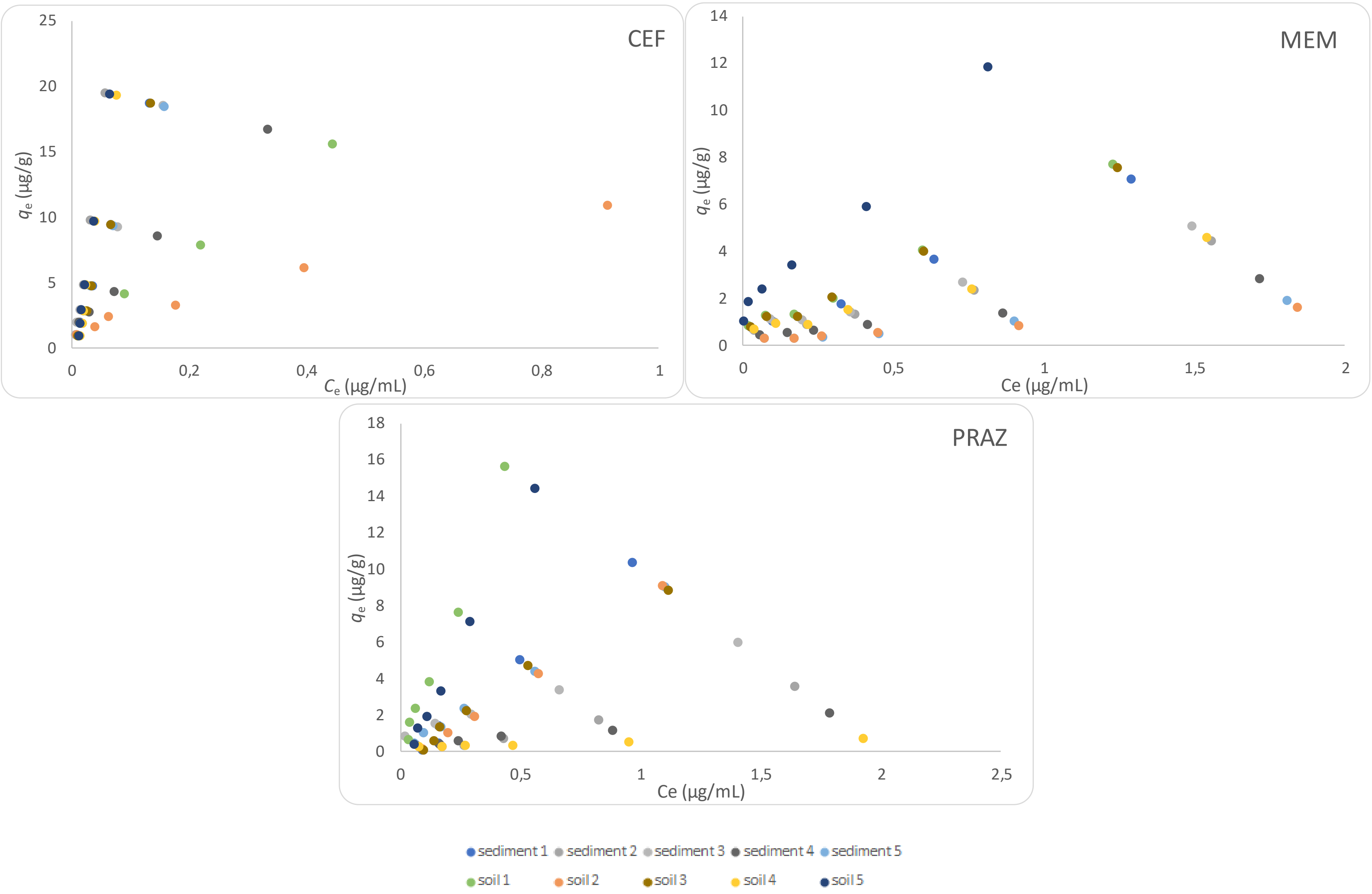

Figure S2 Linear sorption isotherms of CEF, MEM and PRAZ in five sediment samples and in five soil samples at 25 °C.

Table S1. Selected pharmaceuticals, their structures and physico-chemical properties [33]

	Chemical Structure	CAS no.	M_w	pK_a	log K_{ow}
CEF	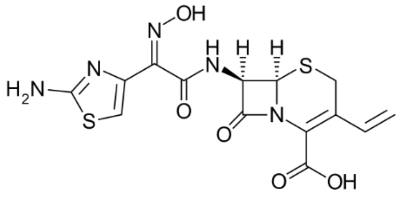	91832-40-5	395.41	9.70	
MEM	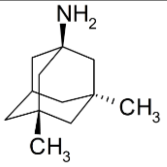	19982-08-2	179.31	10.7	3.28
PRAZ	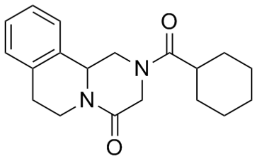	55268-74-1	312.41	n.a.	2.42

Table S2. Physico-chemical characterization of sediment and soil samples

Parameter	Sediment Samples					Soil Samples				
	1	2	3	4	5	1	2	3	4	5
Coarse sand, wt.%	11.10	14.50	15.80	60.35	33.21	45.20	56.65	42.00	40.40	38.31
Clay, wt.%	0.05	0.05	0.05	0.05	0.09	0.10	0.05	0.05	0.05	0.057
Silt, wt.%	0.25	0.25	0.20	0.10	0.24	0.15	0.10	0.30	0.25	0.16
Fine sand, wt.%	88.60	85.20	83.95	39.50	66.45	54.55	43.20	57.65	59.30	61.47
pH	6.97	7.19	7.15	7.22	3.92	7.09	7.25	6.37	5.75	6.00
EC/μS/cm	115.50	127.40	117.20	141.50	26.4	115.30	44.60	149.80	111.90	144.30
TDS*, mg/L	73.92	81.54	75.00	90.56	16.9	73.79	28.54	95.87	71.62	92.40
CEC, mmol/kg	46.48	30.87	54.41	32.07	8.34	51.06	15.78	86.48	44.81	88.70
OM, wt.%	3.00	1.35	2.51	1.09	2.77	0.06	1.52	3.95	3.08	11.59
$CaCO_3$, wt.%	0.44	8.63	3.00	3.75	0.0003	9.40	79.71	0.44	0.063	0.0003
Zn, mg/kg	5.76	4.86	2.21	5.33	4.39	0.85	0.66	2.41	2.95	38.44
Cu, mg/kg	5.54	5.07	5.90	4.73	5.50	1.60	1.25	8.40	5.25	23.18
Fe, mg/kg	116.06	142.92	106.79	134.30	79.70	20.21	24.56	83.05	143.91	304.69
Mn, mg/kg	60.39	26.69	52.46	44.35	21.80	9.54	22.57	35.16	37.97	27.52

TDS* = Total Dissolved Solids
